# Electricity

**Published:** 1880-10

**Authors:** 


### Editorial.

### ELECTRICITY.

Like all other potent therapeutic measures, electricity is often over rated or abused. In no more striking instance do we find this fact illustrated than in the extensive tendency that exists to misapply this powerful and subtile agent in the treatment of disease.

With an almost utter indifference to the detailed principles of its action and application, many there are who, with a general idea of the tonic effect to nerves, as the result of electro-therapeusis, "shock" patients indiscriminately, and often induce decided pathological aggravation. Should agravation not result, there are cases in which no benefit whatever will ensue, unless the special electro-therapeutic indication is understood and comprehended. We find illustration of this fact in occasional cases of facial neuralgia, where one or the other, either faradic or galvanic currents are indicated, and in which the benefit depends upon the proper conception of the indications.

In the dawning era of electro-therapeutics, the frictional or statical electricity was the sole medium of employing this agency. That this has become almost absolute, is reasonable when we consider how much more convenient and generally efficacious is its successors, the galvanic and induced electricity.

In 1791 Galiani discovered the principle of galvinism. He shewed that in the formation of a galvanic circle three elements were necessary of which two were usually metals and the third some liquid. A current, generated by these elements, takes a specific direction, and a proper conception of this fact is of the greatest importance in its therapeutic bearing. An axiom of no small moment in electro-therapeusis is "that where nerve tissue is subjected to the influence of a galvanic current it is variously modified, according to the position of the poles." That is, when the galvanic current flows in the same direction with the nerve current, the strength of the latter is increased. When in a contrary direction it is diminished. The extent to which these principles obtain depends upon various conditions, such as the quantity and tension of the electric current, the length of the nerve effected, and so forth.

The intensity and direction of an electric current depends upon the electrical relation of the generating elements. When zinc and copper with diluted sulphuric acid are used, the current, under the liquid, is from the + or positive zinc, to the — or negative copper; out of the liquid the current is from the copper to the zinc. So that the positive pole of the battery, applied at the sacral, and the negative at the peroneal region, would transmit the electrical along the direction with the nerve current.

The discovery of inductive electricity by Faraday, in 1831–2, instituted a new era in electro-therapeusis. While Remak, of Germany, was enthusiastically lauding the superior benefits of galvanism, Duchenne, of France, entered the lists as an equally enthusiastic champion in behalf of faradization. Remak resorted to the motor nerves as a medium of the galvanic application, while Duchenne selected the muscles for the medium of administering the faradaic current. Both, in the controversy, were dogmatic and extravagant, yet, by their experiences and researches, contributed much to the advancement and enlightenment of a subject that was attended with no little mystery at that time. Through the natural course of events this controversy, kept up by the disciples of the original champions, has finally established a series of distinctive individual indications which are to regulate the application of the Faradic and galvanic currents respectively.

From a physiological standpoint, various experiments have demonstrated the distinctive advantages that the galvanic current possesses over the Faradic, on account, probably, of its greater continuousness of action, in its superior chemical electrotonic, catalytic or electrolytic powers.

In connection with this advantage may be mentioned its greater power of overcoming resistance and its capacity to produce contractions of muscles in instances when the faradic fails. On the other hand, the faradic current produces greater mechanical effects, owing to its rapid interruptions, which excites a double contraction of the muscle, viz: at the opening and closing of the circuit. By its incautious or ignorant application it is less likely to produce serious harm than the galvanic, under the same circumstances.

To sum up, then, the destructive indications for the galvanic current exist "where a *special electrolytic or catalytic power* on any part of the central or peripheral nervous system is desired; where paralyzed muscles have failed to respond to the faradic current;" and where it is desired to produce cauterization.

The differential indication for the faradic current is where mild electrotonic action on the central or peripheral nervous system is desired; to excite muscular contractions rapidly; and to produce strong mechanical effects.

Therefore, to promote more perfectly absorption or catalytic results, the galvnnic is preferable to the faradic. On the other hand, to produce mild electrical or stimulant effects in functional nervous disturbances the faradic current claims the preference. R. W. W.

